# Using repeated lysis steps fractionates between heterotrophic and cyanobacterial DNA extracted from xenic cyanobacterial cultures

**DOI:** 10.1093/g3journal/jkaf135

**Published:** 2025-06-11

**Authors:** Alexis D Wagner, Mohammed M A Ahmed, Victoria A Starks, Paul D Boudreau

**Affiliations:** Department of BioMolecular Science, School of Pharmacy, University of Mississippi: Faser Hall, University, MS 38677, USA; Department of BioMolecular Science, School of Pharmacy, University of Mississippi: Faser Hall, University, MS 38677, USA; Department of Pharmacognosy, Al-Azhar University, Cairo 11371, Egypt; Department of BioMolecular Science, School of Pharmacy, University of Mississippi: Faser Hall, University, MS 38677, USA; Department of BioMolecular Science, School of Pharmacy, University of Mississippi: Faser Hall, University, MS 38677, USA

**Keywords:** cyanobacteria, whole genome sequencing, xenic cultures, microbiomes

## Abstract

Extracting DNA from cyanobacteria can be a challenge because of their diverse morphologies, challenging cellular structure, and the heterotrophic microbiome often present within cyanobacterial cultures. As such, even with high DNA yields, the percentage of reads coming from the cyanobacterial host can be low, leading to an incomplete cyanobacterial genome assembly. In this research, we optimized a DNA isolation protocol using three iterative cell lysis steps to enrich the portion of DNA isolated coming from the cyanobacterial host rather than the heterotrophic microbiome. In order to utilize in-house nanopore sequencing, we faced a challenge using our lysis protocol: the iterative lysis approach led to more DNA shearing than is ideal for this sequencing technology. To solve this, we used two bead-based size selection steps to remove shorter molecules of DNA before nanopore sequencing. Analysis of the sequenced reads showed that, in the first lysis, the cyanobacterial sequences were only 35% of all reads. In the repeated lysis steps, however, the proportion of reads coming from the cyanobacterium increased to 75% or higher. Using our iterative lysis protocol, we were able to sequence the genomes of two fresh water cyanobacteria isolated from northern Mississippi, namely *Leptolyngbya* sp. BL-A-14 and *Limnothrix* sp. BL-A-16. The genomes of these isolates were assembled as closed chromosomes of 7.2 and 4.5 Mb for BL-A-14 and BL-A-16, respectively. As it is not always possible to prepare axenic cultures of cyanobacteria, we hope our approach will be useful for sequencing other xenic cultures of cyanobacteria.

## Introduction

Found in diverse environments around the world, cyanobacteria are well-known for their production of a vast array of secondary metabolites, including many leads in drug discovery efforts (Tan 2007; Gerwick and Moore 2012; Dittmann *et al.* 2015). Due to the diversity of these chemical scaffolds, cyanobacteria are of interest in the biotechnological, industrial, agricultural, and pharmacological fields (Rastogi and Sinha 2009). Although they have many beneficial applications, they are also known producers of harmful algal blooms that can lead to ecosystem destruction, wildlife death, and human health impacts (Hoagland *et al.* 2002; Dodds 2006; Dyson and Huppert 2010; Berdalet *et al.* 2016). In order to better understand the metabolic capabilities and natural product potential of these organisms, there is a great deal of interest in whole genome sequencing of cyanobacteria (Dittmann *et al.* 2015; Leao *et al.* 2017). These genomic datasets can be mined for biosynthetic gene clusters responsible for natural product production because these clusters are often highly organized within a genome and, even for diverse chemical scaffolds, often share common core biosynthetic genes (Jones *et al.* 2010; Blin *et al.* 2023). However, cyanobacteria have multiple features which make them challenging substrates for DNA isolation: They grow with different morphologies, some are single celled, some are quasimulticellular filaments that form macroscopic assemblages (Castenholz and Waterbury 1989; Dvořák *et al.* 2015). Cyanobacteria also possess specialized plant-like cell walls that cause difficulty for traditional chemical lysis protocols and produce enzyme-inhibiting secondary metabolites which can contaminate the DNA, these are both challenges for some common protocols and commercialized bacterial DNA isolation kits (Castenholz and Waterbury 1989; Billi *et al.* 1998; Tillett and Neilan 2000; Wu *et al.* 2000; Morin *et al.* 2010). Their large cells (large relative to most bacteria) also serve as a scaffold for a microbiome community of heterotrophic bacteria, the DNA of which will also be collected in most workflows (Paerl 1982; Billi *et al.* 1998; Cummings *et al.* 2016; Moss *et al.* 2018). A likely role of this heterotrophic microbiome is shared metabolism and exchange of nutrients (e.g. syntrophy around nitrogen fixing, by the heterotrophs or the cyanobacterial partner), however, because few of these associated heterotrophs are well-studied, such codependences are largely speculative (Hube *et al.* 2009; Cummings *et al.* 2016; Hughes *et al.* 2018). Once DNA is sequenced, cyanobacterial genomes can be challenging targets to assemble, with large variance in pangenome size within a given cyanobacterial clade (Dvořák *et al.* 2023), numerous repeat regions (Kaneko *et al.* 2007; Leao *et al.* 2017), or no complete assemblies in closely related strains to guide assembly efforts (as exemplified in this work).

There are DNA extraction protocols that have been developed for cyanobacteria specifically, but they are often targeted for one specific morphology or species (Gaget *et al.* 2017). These protocols come with their own challenges: Approaches using phenol/chloroform-based DNA extractions are popular (Billi *et al.* 1998; Tillett and Neilan 2000; Wu *et al.* 2000; Saha *et al.* 2005; Schober and Kurmayer 2006; Morin *et al.* 2010; Gaget *et al.* 2017); but this mixture is highly toxic, making these methods less desirable (especially for teaching labs with undergraduates), and much research effort has been expended to avoid these reagents (Miller *et al.* 1988; Johns Jr and Paulus-Thomas 1989; Mahuku 2004; Rivero *et al.* 2006; Ghaheri *et al.* 2016; Pérez-Carrascal *et al.* 2021). In this research, we worked to develop a DNA extraction method that was useful even in the presence of a complex microbiome of heterotrophic bacteria. To do so we adapted the Omega Bio-Tek E.Z.N.A. Plant DNA Kit, using repeated, increasingly severe, lysis steps. In sequencing these different DNA fractions separately, we show that the cyanobacterial cells survive (or at least their DNA is not easily recovered in) the initial lysis step, allowing fractionation from heterotrophic bacterial DNA. From these targeted fractions, there was sufficient coverage of the cyanobacterial genomes to allow their assembly.

## Materials and methods

### General experimental equipment and materials

The autoclave used for sterilization was a Steris AMSCO C Series Remanufactured Small Steam Sterilizer. Long-term storage of samples was done at ultra-low temperatures in a VWR Ultra-Low Temperature Upright Freezer set to $-70^{\circ }$ or a or a Thermal-Kool walk in freezer set below $-20^{\circ }$. The LED grow lights from Gardener’s Supply Company, with height adjusted for cultures to receive 1,550 lux on a 16:8 light/dark cycle timer. A ZEISS Widefield Axio microscope within the Glycoscience Center of Research Excellence (GlyCORE) Imaging Research Core was used to image the cyanobacterial and algal cultures. The lyophilizer used was a Labconco Freeze Dry System/Freezone 2.5. The 50 mL tubes were centrifuged in a Sorvall Legend XTR Fixed Angle Centrifuge. The microcentrifuge used was a Sorvall Legend Micro 21R Microcentrifuge. The heat block used was a Fisherbrand Mini Dry Bath. The vortex used was a Fisherbrand Vortex Genie 2. An Invitrogen Qubit 4 Fluorometer was used in accordance with the manufacturer’s Qubit 1× dsDNA High Sensitivity Assay protocol. A Thermo Scientific mySPIN 6 Mini Centrifuge was used to spin down samples in the 1.5 mL microcentrifuge tubes. The nanodrop was a Thermo Scientific NanoDrop OneC.

The petri dishes used were 15×100mm Fisherbrand Petri Dishes with Clear Lid. The 50 mL polypropylene conical natural centrifuge tubes were Falcon brand. The inoculation loops used were Fisherbrand Disposable Inoculating Loops and Needles, Flexible Needle and Loop. The 1.5 mL microcentrifuge tubes used were VWR Polypropylene Microcentrifuge Tube. For column binding, the Zymo Research DNA Clean and Concentrator-5 kit was used according to the protocol from the manufacturer for genomic DNA (>kb). The isopropanol (IPA) used was LabChem Isopropyl Alcohol, LCMS grade. The ethanol used was Decon Laboratories 200 proof ethanol, anhydrous that meets USP specifications. Sera-Mag Select (Cytiva, product number 29,343,045) beads were used following the left side size selection protocol from the manufacturer. AMPure XP beads for DNA cleanup in the nanopore sample preparation came from Beckman Coulter. Chemicals were purchased from high quality lab suppliers, specifically, the germanium (IV) dioxide, ≥99.99% trace metals basis, was from Aldrich, while the nystatin was from Alfa Aesar.

### Isolation of cyanobacteria, collection of cyanobacterial cells

Fresh water (FW) BG-11 plates were made according to a recipe adapted from the University of Texas at Austin Culture Collection of Algae’s BG-11 media recipe (Starks 2022). After autoclave sterilization, 1 L of the agar medium was allowed to cool, but not solidify, then the following were added: 200μL of a filter sterilized 20 mg/mL stock solution of germanium dioxide in water and 1 mL of a 50 mg/mL stock solution of nystatin in 100% ethanol.

Cyanobacterial strains were isolated from fresh water sources in Northern Mississippi. BL-A-14 was from an Enid Lake water sample (assigned BioSample number SAMN42824079); BL-A-16 was from a water sample from the Tennessee-Tombigbee Waterway (assigned BioSample number SAMN42824080). Water samples were collected in sterile 50 mL centrifuge tubes, placed on ice, and then brought to the lab. Fifty microliters from each water sample were then plated directly. Fifty microliters of a 10× concentrated sample were also plated, this concentrated sample was prepared by centrifugation of 1,000μL of the water sample at 21,000 g at 13^∘^ for 5.0 min, removal of the top 90% of the water sample by pipette, and finally vortexing the remaining volume.

All plates were placed under the grow lights for 1–2 months and observed for cyanobacterial colonies that were large enough to be picked. Once colonies appeared, they were restruck under sterile conditions using fresh plates, colonies were picked based on differences in morphology. This process was repeated until a single cyanobacterial morphology was observed on the plate. Then, from that single morphology plate an isolated single colony was picked and inoculated into a 125 mL autoclaved Erlenmeyer flask that contained 50 mL of liquid FW-BG-11 medium. Liquid cultures were grown as continuous cultures, with circa 2 months between passages, using 1 μL/mL inoculation into 50 mL of fresh media.

Cultures were harvested after the accumulation of a substantial amount of biomass, before bleaching or yellowing was observed. Once the culture was ready for harvesting, it was poured in to a 50 mL centrifuge tube and centrifuged for 15 min at 10,000 g at 13^∘^. After centrifugation, the biomass accumulated as a pellet was then frozen at ultra-low temperature. The frozen pellet was then lyophilized overnight. Once the pellet was fully dried, it was either placed back into the ultra-low temperature freezer for storage until use, or used immediately. Harvests of 50 mL cultures had variable yields, ca. 30–70 mg dry weight.

### DNA isolation for illumina sequencing

For BL-A-14, after collecting cyanobacterial pellets from 50 mL cultures, gDNA isolation was performed using the Omega Bio-Tek E.Z.N.A. Plant DNA kit according to the manufacturer’s instructions. In brief, the lyophilized cyanobacterial cells underwent lysis employing P1 buffer (with 65.0^∘^ incubation for 10 min), while the P2 buffer was utilized to precipitate proteins and polysaccharides, 70% isopropanol precipitation, redissolving the pellet in 300 μL of $55^{\circ }$ MilliQ water. Subsequently, the resulting clear supernatants were then bound to a HiBind column, washed with the manufacturer’s buffers, and eluted using 50 μL of $55^{\circ }$ MilliQ water incubated at room temperature for 5.0 min then a combined second elution of 50 μL of $55^{\circ }$ MilliQ water collected immediately. For BL-A-16, the DNA was isolated using Omega Bio-Tek E.Z.N.A. Bacterial DNA kit following the manufacturer’s protocol. Briefly, we used the optional bead beating by vortexing with ca. 25 mg of glass beads for 5 min after the initial heating with lysozyme at 37^∘^ for 20 min. After the bead beating with TL buffer, the sample was heated at 55^∘^ for 1 h (vortexing briefly to mix every 20 min), the DNA was collected with elutions of 75 μL of 65^∘^ warmed MilliQ water incubated for 5.0 min, then 50 μL incubated for 2.0 min, and then 50 μL eluted immediately. DNA quantification was carried out via nanodrop before shipping for sequencing, where DNA concentration was insufficient for the vendor (in the case of BL-A-16), the material was concentrated using Zymo’s DNA Clean & Concentrator-5 kit according to the manufacturer’s protocol. This material was sent for Illumina MiSeq 2×150 bp sequencing with a commercial vendor (Genewiz).

### Iterative lysis protocol for DNA isolation targeting cyanobacterial DNA

Two lyophilized pellets from 50 mL harvests (of two separate passages) of the desired strain were used for the DNA isolation. An adapted version of the Omega Bio-Tek E.Z.N.A. Plant DNA Kit protocol was followed: First, the two dried cell pellets were combined and 1,000 μL of P1 buffer was added to the pellets, this mixture was vortexed briefly. Once vortexed, the 50 mL centrifuge tube was placed in a 60–65^∘^ water bath for 10 min. After the 10-min incubation, the lysate was transferred to a 1.5 mL microcentrifuge tube and centrifuged at 21,000 g at 13^∘^ for 2.0 min in the microcentrifuge. Once centrifuged, the supernatant was collected by pipette and placed in a separate sterile 1.5 mL microcentrifuge tube.

With the pellet from the previous step, 800 μL of P1 buffer, 10 μL of lysozyme (50 mg/mL), 10 μL of Proteinase K (20 mg/mL) and ca. 10–20 mg of glass beads (the P1 buffer came from the Omega BioTek E.Z.N.A. Plant DNA Kit while the other materials came from Omega BioTek E.Z.N.A. Bacterial DNA Kit) were added to the sample; we then vortexed this mixture briefly. The vortexed sample mixture was then placed in the preheated heat block at 65^∘^ for 20 min; to mix the tube, we inverted the tube six times halfway through the incubation. Then the supernatant was collected as before. The second pellet was incubated with 800 μL of P1 buffer, 10 μL of lysozyme, and 10 μL of Proteinase K for 1 h at 65^∘^ and inverted six times twice, at the 20- and 40-min points. After the incubation, the sample was centrifuged as before, and the final supernatant was collected.

With the supernatants from the previous steps, these samples were either stored on ice, for a maximum of 1 h, or used immediately. To these samples, 140 μL of P2 buffer was added and inverted six times to mix. The mixture was then centrifuged at 21,000 g at 13^∘^ for 10 min in the microcentrifuge. Once centrifuged, the supernatant (ca. 200–800 μL has been recovered, note that low volume at this step was not observed to interfere with DNA recovery) was collected in a sterile 1.5 mL microcentrifuge tube and a volume of IPA equal to 70% of the mixture’s volume was added. The tube was then inverted six times to mix before centrifuging for 5 min at 21,000 g and 13^∘^ in the microcentrifuge. Once centrifuged, the supernatant was discarded and the pellet was collected. Next, 300 μL of 65^∘^ MilliQ water was added to the pellet, to aid the pellet in dissolving, it was placed in the preheated 65^∘^ heat block for 10 min. After the incubation, 4.0 μL of RNase (10 mg/mL, Omega Bio-Tek) was added and the DNA was measured on the Qubit with 2.00 μL of the sample.

Next, the samples were cleaned and concentrated during the column binding step using the Zymo DNA Clean and Concentrator-5 kit in accordance with the protocol for genomic DNA from the manufacturer. For the elution steps, we first specifically used 17.0 μL of sterilized MilliQ added directly to the column matrix and then incubated at room temperature for 5 min before collecting this eluent by centrifugation for 30 s at 21,000 g and 13^∘^. The second elution was then performed with 15.0 μL of sterilized MilliQ water and a 3.0-min incubation, also at room temperature, before being combined with the first by centrifugation. The column was then discarded, and the sample was measured on the Qubit as mentioned above. Once measured, the sample was frozen at $-70^{\circ }$ for storage.

### Bead-based size selection of DNA before nanopore sequencing

Following the Sera-Mag Select (Cytiva) left side size selection protocol, we added beads at a 2:1 ratio to the sample volume. Next, the 1.5 mL microcentrifuge tube was flicked for 60 s to mix and avoid shearing DNA before being briefly spun down and incubated at room temperature for 7 min. The tube was then placed on the magnetic rack for 5 min to pellet the beads fully. Then the supernatant was pipetted off and discarded. The beads were washed twice, leaving the tube on the magnet, with 200 μL of freshly prepared 85% ethanol, taking care not to disturb the pellet when removing the wash solution by pipette. After the second wash solution was removed, care was taken to remove any remaining drops of the wash solution by pipette, before air drying the beads on the magnet for 7 min at room temperature. The microcentrifuge tube was removed from the magnetic rack and 50 μL of Tris-EDTA buffer was added to elute the DNA. The Sera-Mag Select beads were resuspended by flicking the tube for 60 s, the tube was then briefly spun down before being incubated for 7 min at room temperature. After incubation, the microcentrifuge tube was placed in the magnetic rack for 5 min to fully settle the beads. Once settled, the supernatant was carefully pipetted off and transferred to a new sterile microcentrifuge tube.

The entire Sera-Mag Select size selection protocol was repeated a second time on this bead-purified material and then the final recovered DNA was measured on the Qubit as before. The sample was then frozen at $-70^{\circ }$ until use.

### Genome sequencing via the nanopore platform

Doubly size selected DNA was sequenced on an Oxford Nanopore Flongle cells (FLO-FLG001, R9.4.1 chemistry) using the Ligation Sequencing Kit (SQK-LSK100) according to the manufacturer’s protocol. The end-prep and nick repair were conducted without deviation from the protocol using 500 ng of the doubly size selected DNA. Next during the adapter ligation, again the manufacturer’s protocol was followed using New England Biolab’s Quick T4 DNA Ligase, however, during the cleanup step instead of washing the AMPure XP beads with 125 μL of Long Fragment Buffer or Short Fragment Buffer, we first washed with 125 μL of Long Fragment Buffer then 125 μL of Short Fragment Buffer for the second wash. During the final loading of the Flongle, reagents in glass vials were used from the Flongle Sequencing Expansion, following the manufacturer’s protocol. In setting the run parameters, we selected our flow cell, kit, and a 48-h total run time.

### Bioinformatic processing and analysis of DNA read data

Illumina data were processed by the commercial vendor; briefly, after a FastQC quality check de novo genome assembly was carried out with Spades v 3.10 and genome statistics were generated via QUAST. No filtering or trimming on the reads was done as these initial assemblies were disjointed across numerous contigs, as such we did not value improving the Illumina-based assemblies, but rather using the raw read sets to polish our later nanopore-based assemblies. To assess origin of contigs within these initial draft Illumina-based assemblies, in Geneious Prime (version 2023.0.4), the first 1,000 bp was extracted from all contigs in both assemblies and a Megablast search was run against the nucleotide collection (nr/nt) (Supporting Information, Supplementary File S1—Supplementary Table E) (Zhang Z *et al.* 2000; Morgulis *et al.* 2008).

After the nanopore sequencing run, data were transferred to the computing resources in the GlyCORE Computational Chemistry and Bioinformatics Core for processing and assembly of the genomes. The first step of processing the sequencing reads was to basecall the .fast5 nanopore reads using Guppy basecaller (version 6.4.2), for which we used the super accuracy configuration file for our kit and flowcell. Once the reads were basecalled to .fastq files, a pycoQC (version 2.5.2) report was performed to assess the quality of reads (Leger and Leonardi 2019).

We used the Porechop tool (version 0.2.4) to remove adapter sequences from the raw basecalled reads (Wick 2018). Next, these adapter-free sequences were run through the FiltLong tool (version 0.2.1) (Wick 2021). In the FiltLong settings, we put the length cutoff threshold to the tenth percentile as determined by the pycoQC report or 500 bp, whichever was shorter. We set the quality score to filter out at least 1% of reads by quality, but as this step occurs after filtering by length, reads were effectively only filtered by length.

To assess fractionation of heterotrophic and cyanobacterial DNA, we used the EPI2ME and Geneious Prime mapper tools. The EPI2ME analysis was conducted with version 5.2.3 of the software available from Oxford Nanopore (Oxford Nanopore Technologies 2023). We submitted our FiltLong filtered .fastq files to the Metagenomics workflow (v2.10.1) analysis.

Separately, the Geneious Prime software platform (version 2023.0.4) was used to map the FiltLong filtered reads of each flongle run to the full assembled genome (plasmids included) of their respective cyanobacterium. For this analysis, Geneious’ Mapper was used with the sensitivity setting of “Medium Sensitivity/Fast” using no fine tuning or trimming. Total number of reads mapped to the genome was reported from the saved list of used reads. This analysis was repeated with the vendor’s Illumina-based scaffolds to assess the contigs within this assembly as being from the cyanobacterium vs the heterotrophic microbiome. A separate run was conducted with the Illumina derived scaffolds mapping exclusively to the cyanobacterial chromosomes to create an Illumina chromosomal sequence set for comparison against the nanopore-based assembly.

### Genome assembly, statistics, and comparison

Once Filtlong processing was complete, an initial draft assembly was constructed with Flye using the metagenomic setting (version 2.9-b1778) (Kolmogorov *et al.* 2019). Medaka (version 1.7.2) was used to improve the draft Flye assembly [50], with the run inputs being the draft Flye assembly and the combined reads files of all fractions after FiltLong trimming, the default batch size was 500 using model r941_min_sup_g507. After Medaka long-read polishing, the Polypolish tool (v0.5.0) was used to correct any of the long-read assembly errors with previously collected Illumina data, to confer the benefits of long-read assembly (large contigs) with the benefits of short-read assembly (high accuracy) (Wick and Holt 2022; Wick *et al.* 2023). Polypolish was coupled with the BWA-MEM2 (Burrows-Wheeler Alignment) tool (version 2.2.1_x64) (Vasimuddin *et al.* 2019), which was used to index the Illumina reads to the Medaka-corrected consensus assembly as a .sam alignment. Polypolish was then run using the Medaka consensus assembly file, and the two filtered .sam files to produce a polished assembly of our sequenced genomes.

DFAST annotation was done with the online version of the tool available from the DNA Data Bank of Japan, National Institute of Genetics (DNA Data Bank of Japan 2018; Tanizawa *et al.* 2018). In this project, we ran the DFAST tool on our circular chromosomes of cyanobacterial DNA extracted from our metagenomic assemblies as well as the Illumina draft assembly scaffolds that mapped to the final cyanobacterial chromosome.

We used the Type Strain Genome Server (TYGS) to analyze our cyanobacterial chromosomes and compare them to the TYGS’s database, based on both 16S and whole genome-based phylograms (Meier-Kolthoff and Göker 2019). Each polished chromosome was uploaded to the server as a separate job, see Supporting Information for additional details on the methods used by the TYGS platform, with the genome phylogram results presented in the Supporting Information (Supporting Information, Supplementary File S1—Methods Supplement and Supplementary Figs. I and J).

The OrthoANI Tool (OAT) analysis was run with version 0.93.1 of the tool (Lee *et al.* 2016). All chromosome or complete assemblies on GenBank in the genera *Leptolyngbya* or *Pseudanabaena* were downloaded and the chromosome contig was extracted from each. With the OAT, this data set was compared to our chromosomes in two batches. Settings were for the use of two threads, with the original ANI calculated in both directions managing two different values by averaging them, Genome-to-Genome Distance Calculator set to Form 2, and calculating the OrthoANI. The heatmap of this analysis is presented in the Supporting Information (Supporting Information, Supplementary File S1—Supplementary Fig. K).

Finally, the other contigs present within the assembly were analyzed. Two contigs across the assemblies BL-A-14-contig_10 and BL-A-16-contig_7 were nearly identical to each other and had a >99% pairwise identity with *E. coli* strain Q4552 plasmid pECQ4552_IHU08 (CP077071) as assessed using a blastn search in Geneious prime (version 2023.0.4). Given this similarity across to the two assemblies, these contigs were deemed contaminants from our DNA isolation workflow; note that the coverage of these contigs was also far higher than any other contig in the assemblies (Supporting Information, Supplementary File S1—Supplementary Tables A and B). In the assembly of BL-A-14, 34 contigs between 1,216 and 6,561 bp were binned out by their high GC content (ranging from 68.2% to 76.2%). Again, blastn analysis showed the nearest hits to these nucleotide sequences were not cyanobacterial sequences, instead these hits were all Actinobacteria, suggestive that these contigs came from a heterotrophic member of *Leptolyngba* sp. BL-A-14’s microbiome. The remaining seven contigs were assembled as circular contigs between 2,182 and 288,243 bp, with GC content far closer to the cyanobacterial genome (ranging from 48.3% to 52.0%), and blastn analysis of open reading frames on these contigs showed the most similar sequences were mostly cyanobacterial. As such we assigned these seven contigs as plasmids of this strain. Unsurprisingly, based on our EPI2ME analysis, we found several contigs within the assembly of BL-A-16 that were more similar to *Pseudomonas*. Binning by GC content pulled five contigs out of the assembly, with lengths of 182,682 to 1,612,854 bp and GC content ranging from 62.4% to 63.6%. Blastn analysis of open reading frames within these contigs confirmed them as being *Pseudomonas*-derived, we even found three identical copies of the 16S gene which had a 100% pairwise identity to the full 16S gene of *Pseudomonas argentinensis* strain AFS0086324 (OP986251). Two of the remaining contigs were assembled as circular by Flye, BL-A-16-contig_10 had a length of 55,181 bp and a GC content of 55.3% while BL-A-16-contig_11 had a length of 15,919 bp and GC content of 53.0%. We assigned these sequences as cyanobacterial plasmids based on their GC content and circular topology. Blastn analysis of open reading frames from within BL-A-16-contig_11 confirmed this assignment with hits to other cyanobacterial sequences, while BL-A-16-contig_10 showed few hits to any known sequences in GenBank (cyanobacterial or otherwise). The last contig was a linear 5,804 bp fragment with 51.3% GC which had no blastn hits to known sequences so this fragment was removed from the assembly.

Following up this work to characterize the short contigs with a geNomad analysis, the polished genomes were submitted to an analysis using version 1.11.0 of the program (Camargo *et al.* 2024). For BL-A-16, this workflow annotated the 16 kb contig (CP166613.1) in this genome as a plasmid (0.9884 plasmid score), but the 55 kb contig (PV404223) as a virus (0.8927 virus score). In BL-A-14, this workflow annotated the 25 kb contig (CP166616.1) as a plasmid (0.9418 plasmid score) but did not annotate any of the other small circular contigs as plasmids or viruses, so our annotation of the other short circular contigs within the genome of BL-A-14 remains preliminary and will need further characterization.

The polished assemblies of these two species were deposited with GenBank under the BioProject number PRJNA1048477. The assembly of *Leptolyngbya* sp. BL-A-14 was given the accession numbers CP166616-16623 (chromosome as CP166621), while the assembly of *Limnothrix* sp. BL-A-16 was given the accession numbers CP166613 and 16615 (chromosome as CP166615) with the phage sequence deposited separately (PV404223). The FiltLong filtered nanopore reads and the raw Illumina reads are available as sequence read archives on GenBank (SRR32600066-32600072). The full PycoQC reports of the nanopore runs, the scaffolds of the vendor’s Illumina-based assemblies, and the original Polypolished assemblies (before removal of contigs assessed as being from the heterotrophic microbiome by GC content and BLAST similarity) are available on the eGrove archive (see Data availability).

### AntiSMASH analysis

The final cyanobacterial genomes were submitted to the antiSMASH bacterial sequence annotation pipeline (version 7.1.0, using the online platform), with a detection strictness setting of “relaxed,” 10 hits were found in *Leptolyngbya* sp. BL-A-14 while 4 hits were found in *Limnothrix* sp. BL-A-16. To compare this result to the initial Illumina-derived contigs, the Illumina scaffolds were mapped to the final genomes with the Geneious Prime (version 2023.0.4) Mapper tool with the sensitivity setting at “Medium Sensitivity/Fast” and exporting the used reads to a list. The 614 used reads found for BL-A-14 and the 176 used reads found for BL-A-16 were dubbed the Draft Illumina Assemblies, and these assemblies were also submitted to antiSMASH with the same settings as for the final assemblies (Supporting Information, Supplementary File S1—Supplementary Table F).

The antiSMASH annotation for *lim* pathway in *Limnothrix* sp. BL-A-16 was imported into Geneious Prime (version 2023.0.4) and the four limE homologs were translated and aligned against the known limE homologs from *Limnothrix* sp. CACIAM 69d (Zhang Y *et al.* 2022). The NCBI’s Prokaryotic Genome Annotation Pipeline-derived annotations (by NCBI, version 6.9 for BL-A-16 and version 4.6 for CACIAM 69d) of the two *lim* pathways was compared and the genes and predicted protein sequences were aligned to compare sequence identity, for both the gene and protein alignments the MUSCLE PPP algorithm was used (Supporting Information, Supplementary File S1—Supplementary Table G). The annotation for the BL-A-16 *lim* pathway has also been included in the eGrove archive (see Data availability).

### Use of a large language model

During manuscript preparation, ChatGPT (GPT-4-turbo) was used to convert the .tsv tables in the Supporting Information into LaTeX format, with queries such as: “Convert this tsv to Latex format: [copied tsv table].” It was also used to correct the LaTeX formatting of figures and tables to ensure that they placed in the correct place in the final document. Note that resulting tables were checked manually and the large language model was not used to generate text or images within this manuscript.

## Results

### Cyanobacteria were isolated from fresh water sources across northern Mississippi

We collected water samples from water bodies in Northern Mississippi. BL-A-14 was isolated from a water sample taken from Enid lake, while BL-A-16 was isolated from a water sample collected from the Tennessee-Tombigbee waterway. Upon returning to the laboratory the water was plated directly on agar plates for colony isolation, but since the purification of single morphologies of cyanobacteria, they have been grown as liquid cultures in continuous culture in our laboratory.

### Initial whole genome sequencing efforts via illumina led to disjointed assemblies

We prepared DNA extractions from our xenic cultures of cyanobacteria using the commercial E.Z.N.A. Plant or Bacterial DNA kits from Omega Bio-Tek. When submitted to Genewiz (a commercial vendor) for Illumina sequencing we got high quality read data back, but the resulting genomic assemblies contained hundreds of contigs (669 contigs for BL-A-14 and 263 contigs for BL-A-16) with N50’s below one hundred thousand base pairs (21,192 bp for BL-A-14 and 86,514 bp for BL-A-16). Based on sequence comparison to public databases, we believe these sequences come from both the cyanobacterial target and its heterotrophic microbiome (Supporting Information, Supplementary File S1—Supplementary Table E). Though with BL-A-14 over 90% of contigs map to the cyanobacterium, this draft genome assembly was spread across 617 contigs, while in the draft metagenome for BL-A-16, only 180 (68%) contigs came from the cyanobacterium. A result which highlights both the traditional challenge of disjointed assemblies from short read-based approaches (as seen with BL-A-14), and the specific challenge of working with xenic cultures where assemblies can be confounded by the presence of DNA from heterotrophic bacteria (as seen with BL-A-16). This latter challenge is independent from producing DNA extracts of good quality and concentration. The improved recovery of cyanobacterial DNA (as assessed by proportion of cyanobacterial contigs within the draft Illumina metagenomic assembly) with the Plant DNA kit guided the choice of this kit for the iterative protocol.

### Iterative lysis steps isolated DNA with reduced heterotrophic DNA contamination

**Fig. 1.**
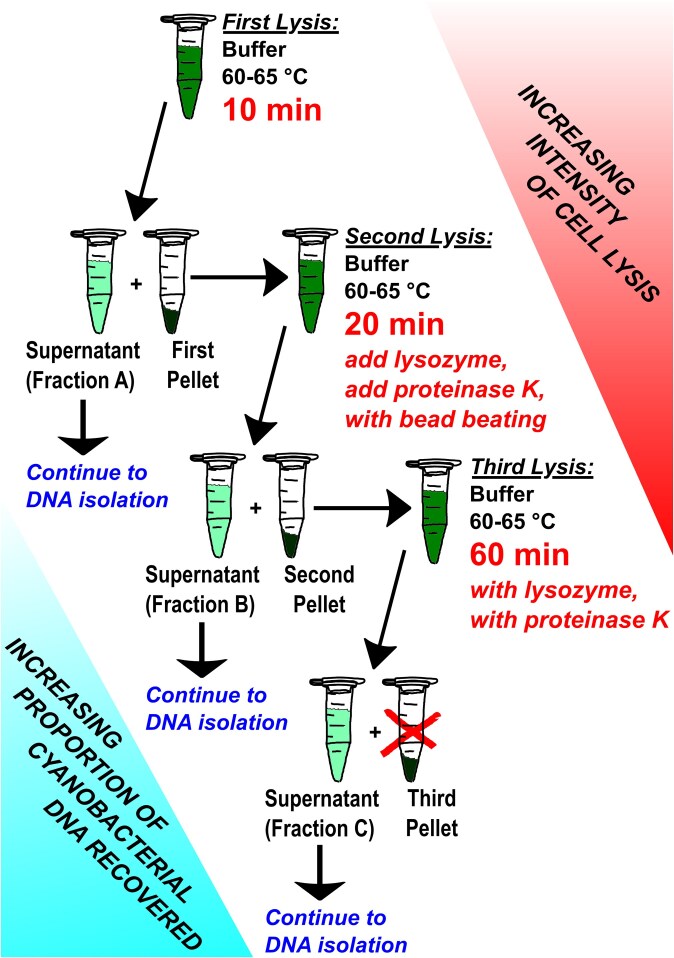
The iterative lysis workflow. The initial lysis step is incomplete, after collecting the pellet there is still DNA available to isolate in the solid cell matter. By repeating the lysis steps more vigorously, more DNA can be collected. In addition, because the cyanobacteria are generally more difficult to lyse than heterotrophic bacteria, the proportion of cyanobacterial DNA increases in each lysis fraction.

**Fig. 2.**
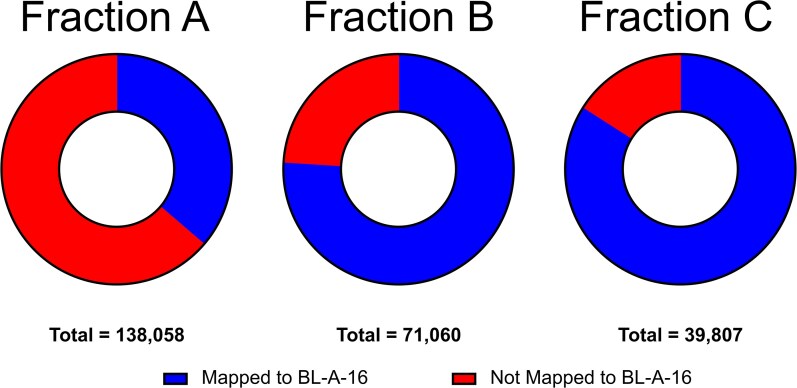
The Geneious mapping of reads from sequencing runs to the final BL-A-16 genome. The proportion of reads from fraction A to B to C mapped to the final cyanobacterial genome shows an increase with each successive fractionation. Which we argue represents the winnowing of heterotrophic DNA from the sample during the iterative lysis. Total refers to total number of reads.

We subsequently prepared three separate DNA samples from our extraction of a cyanobacterial culture, for both BL-A-14 and BL-A-16, again using the Plant DNA Kit. In this preparation, we divided the lysis step into three parts: initially a brief 10 min lysis, then collecting the residual cell pellet, we isolated another lysate from the pellet after 20 min of lysis, and finally collecting a lysate on the residual cell pellet from the previous step utilizing a 1 h lysis; with lysis aids added to the second and third lysis steps (Fig. 1). These three lysates were then processed to isolate three separate DNA preparations for each cyanobacterium. Before a sequencing run using Oxford’s nanopore technology, the DNA had to be size-selected as our lysis product generally lead to significant sheering of the DNA; using two magnetic bead-based size selection washes with Sera-Mag Select (Cytiva) beads we were able to enrich for the longer reads sufficient to run on a nanopore cell (we used flongle cells, FLO-FLG001 with R9.4.1 chemistry). Cyanobacteria are underrepresented in whole genome databases, as reflected by the comparison of 5,634 cyanobacterial genomes to 44,996 Actinomycetes or 214,930 Pseudomonadota genomes in the Genome Taxonomy Database (Australian Centre for Ecogenomics 2024). Furthermore, our BL-A-16 strain is the first complete chromosome assembled in the genus *Limnothrix* available on GenBank (National Library of Medicine, National Center for Biotechnology Information 2024). So, it is not surprising that using the EPI2ME platform, which relies on similarity to known sequences, we saw many of the reads used to generate our assemblies binned out as unknown. However, among the EPI2ME mapped reads, there was a striking decrease in heterotrophic reads coming from the Pseudomonadota after each lysis step on the sample of BL-A-16 (Supporting Information, Supplementary File S1—Supplementary Table H). Conversely, the proportion of reads in the BL-A-16 fractions that were mapped using Geneious Prime to our final cyanobacterial assembly increased from 35% to 75% to 83% with each iterative fractionation (Fig. 2), which supports our hypothesis that our early mild lysis steps are better at popping open heterotrophic cells while the cyanobacteria cells remain intact in the pellet until the later lysis steps.

A control experiment of mapping fraction C from BL-A-16 to the genome of BL-A-14 saw fewer than 1% of reads used, suggesting that any noise from off-target mapping of metagenomic reads to the genome is minimal (Supporting Information, Supplementary File S1—Supplementary Table D). There was little fractionation observed between fractions B and C for BL-A-14. However, both sets of B and C fractions had >75% of reads mapping to the cyanobacterial genome, so we were able to assemble both cyanobacterial genomes. While the fractionation of BL-A-16 does not show increased benefits with each fraction, it does help to show that the fractionation can afford extracts rich in cyanobacterial DNA from a xenic culture.

### Genome assembly using the combined genomic data suggested our strains were novel

We used the Porechop and FiltLong tools to trim and filter our basecalled nanopore reads before assembly with Flye to afford metagenomic assemblies of our cyanobacterium/heterotrophic microbiome communities. In both the assemblies of BL-A-14 and BL-A-16, the largest of the contigs was a closed circular chromosome which, based on BLAST of representative genes (i.e. the 16S gene), we suspected was our cyanobacterial host (Supporting Information, Supplementary File S1—Supplementary Tables A and B). To correct errors in the nanopore sequencing data, we first used Medaka correction with the original nanopore reads and then Polypolish with our earlier Illumina reads to polish this genome (Wick and Holt 2022; Wick *et al.* 2023). DFAST annotation of these cyanobacterial chromosomes showed that the coding ratio increased after polishing while the total number of coding sequences (CDS) decreased (Supporting Information, Supplementary File S1—Supplementary Table C); due to what we believe is correction of errors, such as homopolymer errors, within genes that incorrectly introduced stop codons within a single reading frame leading to two split CDS for a single gene in the early drafts of the assembly. The final number of CDS was similar to the draft assembly of the chromosome derived exclusively from the Illumina data, but with key improvements, such as the assembly into a circular contig rather than over one hundred scaffolds, and the ability to annotate all the rRNA genes (Supporting Information, Supplementary File S1—Supplementary Table C). For BL-A-14, seven circular contigs were also assembled, a geNomad analysis of these other contigs identified no phage sequences and gave the one at 24.5 kb a high plasmid score (0.9418) (Camargo *et al.* 2024). Further examination of these contigs will be necessary, with tentative identification of these contigs as plasmids based on their small size and circular architecture. In the BL-A-16 assembly, the two other circular contigs found were annotated by geNomad analysis to be a circular plasmid (at 15.9 kb) and a circular phage genome (at 55.2 kb) (Camargo *et al.* 2024), with the phage genome then removed from the cyanobacterial assembly.

A TYGS comparison of the BL-A-14 and BL-A-16 genomes suggested each strain was a new species (Meier-Kolthoff and Göker 2019). However, the results were confusing as the TYGS workflow was picking distantly related cyanobacterial strains, and some noncyanobacterial strains (e.g. *Microbacterium aurum* DSM 8,600 in the BL-A-14 analysis) as the closest relatives for its analysis (Supporting Information, Supplementary File S1—Supplementary Figs. I and J). By using a simpler BLAST comparison of the 16S gene extracted from our BL-A-14 chromosome we saw the top hit was to *Leptolyngbya* sp. CENA387 (KR137608), with a 99.6% pairwise identity; for BL-A-16 the same analysis showed a top hit to *Limnothrix* sp. SK1-2-1 (LC272581), with a 99.9% pairwise identity. This comfortably assigned our strain as belonging to the genera *Leptolyngbya* and *Limnothrix*. While there are complete chromosome assemblies for the *Leptolyngbya* genus, they vary in size from 4.4 to 7.2 Mb (see ASM2624063v1 and ASM236825v1), and there are not chromosome or complete level assemblies for other *Limnothrix* strains on GenBank, so we hypothesize that the discrepancy between the TYGS analyses and our 16S-based analysis lies in the difficulty of genomic comparison with large size differences in the genome or with when compared to incomplete assemblies. A subsequent comparison using the OAT showed that our chromosomes had <70% similarity to any chromosome extracted from assemblies that were assembled to the chromosome or complete level which could be downloaded from GenBank within the genera *Leptolyngbya* or *Pseudanabaena* (Supporting Information, Supplementary File S1—Supplementary Fig. K) (Lee *et al.* 2016). Note that our assembly was the only such genome in the genus *Limnothrix* on GenBank, so the genus *Pseudanabaena* was selected because it, like *Limnothrix*, is within the family Pseudanabaenaceae. These results also support the novelty of our genomes for these two organisms and the value in sequencing bacteria like cyanobacteria which are underrepresented in public databases.

### Our genomes suggest the potential for natural product discovery

**Fig. 3.**
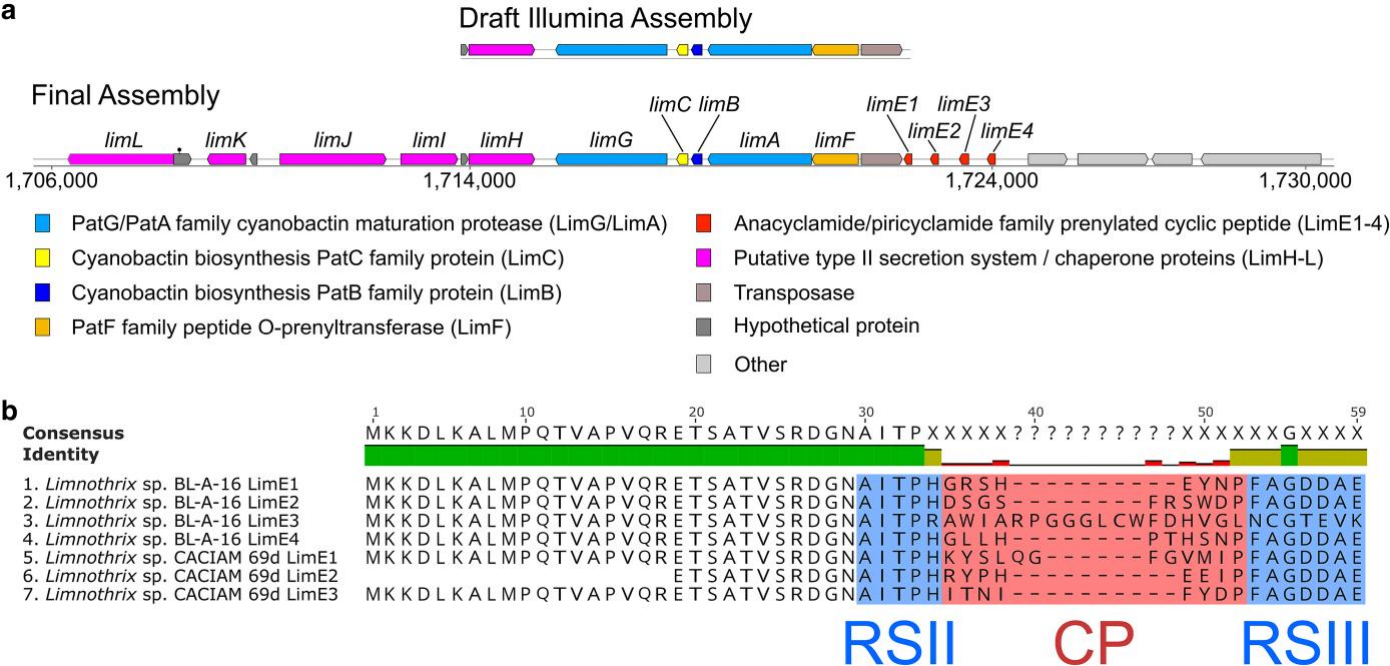
The antiSMASH annotation of the BL-A-16 lim *pathway.* a) The final complete assembly allowed annotation of the full pathway, vs the clipped version from the draft Illumina-derived assembly. In addition to detecting four *limE* homologs, the complete assembly showed a transposase as was seen before in the CACIAM 69d *lim* cluster, Zhang Y *et al.* (2022) but also genes which may be involved in a type II secretion system (e.g. a type II secretion system F family protein gene that we dub *limH*). We hypothesize based on the adjacency of *limH-L* to the core biosynthetic genes that they may be responsible for excreting the final cyanobactins. AntiSMASH annotations of genes and homologs in the *lim* pathway (parentheticals) are given in the color-coded key. b) Comparing the predicted protein sequences for BL-A-16 *limE1*, *limE2*, and *limE4* to the prior *limE1* and *limE3* homologs, discussed by Zhang and coworkers (Zhang Y *et al.* 2022), showed identical leader peptides, recognition sequences for A-family proteases (RSII) domains, and recognition sequences for G-family macrocyclases (RSIII), but different core peptide (CP) sequences. While *limE3* from BL-A-16 had differences in the RSII, CP, and RSIII domains from the other homologs.

Our assemblies were valuable for their completeness (rare in these genera), which allowed comparison of the chromosomes with tools such as the OAT (Lee *et al.* 2016). In addition, with a major motivation for studying cyanobacteria, and their genomes specifically, being the discovery of drug-like molecules (Gerwick and Moore 2012; Leao *et al.* 2017), we also wanted to investigate these genomes for natural product biosynthetic gene clusters. Using the antiSMASH tool (Blin *et al.* 2023), we observed a homolog for the *lim* pathway which antiSMASH scored with a 100% similarity score to the previously reported pathway in *Limnothrix* sp. CACIAM 69d (Zhang Y *et al.* 2022; Blin *et al.* 2023). This result was notable for two reasons: First, a draft assembly derived exclusively from the untargeted single extraction protocol used for the Illumina sequencing led to an assembly which contained this cluster too, but had the *lim* cluster on a contig edge and did not contain the sequence of the *limE* homologs and, as such, would not have allowed prediction of the structures of these natural products (Fig. 3a). Secondly, though the *limE* homologs from BL-A-16 had leader peptide sequences with identity to *limE1*/*limE3* from CACIAM 69d (Zhang Y *et al.* 2022), none of the core peptides matched, suggesting this cluster is responsible for producing novel natural products chemistry (Fig. 3b). In addition, the antiSMASH analysis detected an adjacent set of genes which have conserved synteny between the two clusters, have high percent sequence identity (gene and predicted protein sequence), and by annotations from the NCBI Prokaryotic Genome Annotation Pipeline are putative associated with peptide export/assembly (Supporting Information, Supplementary File S1—Supplementary Table G). Specifically, a type II secretion system F family protein, a type IV pilus twitching motility protein, a GspE/PulE family protein, a GspE nucleotide exchange factor, and a DnaK molecular chaperone that we dub *limH-L*, respectively. These genes were not discussed in the prior work on *Limnothrix* sp. CACIAM 69d (Zhang Y *et al.* 2022) and though they number far short of the 12–15 components within characterized type II secretion systems (Korotkov *et al.* 2012), we argue their conservation within the pathway makes it reasonable to hypothesize they are involved in cyanobactin export. As such, these genes may be crucial to future efforts such as heterologous expression, however, until the molecules are observed *in situ*, their ultimate fate remains unknown.

## Discussion

Our lab’s interest in genomic investigation of cyanobacteria collected from Nature faces some of the hurdles long known in this field, such as the challenge of having heterotrophic DNA contaminate DNA extractions from a cyanobacterial strain serving as host to heterotrophic bacteria (Paerl 1982; Billi *et al.* 1998; Cummings *et al.* 2016; Moss *et al.* 2018 ). Our initial attempts to use Illumina sequencing for our strains was confounded by just this result, leading to incomplete and disjointed assemblies of the cyanobacterial genome. Utilizing a modified lysis approach where we used iterative steps beginning with a quick lysis ill-suited to lyse the cyanobacterial cells, followed by longer lysis steps repeated on the residual cell pellet, we saw heterotrophic reads of the sequenced DNA overrepresented in DNA from the first lysis step while cyanobacterial reads were enriched in later fractions. To derive DNA suitable for nanopore sequencing we had to rely on multiple bead-based size selection steps to enrich for longer molecules, which allowed us to utilize this sequencing technology. However, we believe that the ability to target a specific portion of the microbiome could be broadly applicable to many different sequencing technologies. The remarkable change in proportion of total reads coming from the cyanobacterium in the iterative DNA extracts of *Limnothrix* sp. BL-A-16, from 35% in fraction A to 83% in fraction C provides clear evidence that our strategy can help pull apart complex microbiomes during the DNA extraction step. Other researchers interested in cyanobacterial genomics could similarly apply this workflow to their own projects. As our results with BL-A-14 showed minimal changes in cyanobacterial reads between fractions B and C, but good recovery of cyanobacterial DNA, the need to validate this approach across the broad morphological diversity of cyanobacteria is a clear next step. Both BL-A-14 and BL-A-16 have a quasimulticellular filamentous morphology (Supporting Information, Supplementary File S1—Methods Supplement and Supplementary Figs. L and M), so next targets might include strains with other morphologies, e.g. single-celled cyanobacteria or strains with branching filaments. By purposely sequencing the fraction A we may also be able to investigate the composition of the microbiome which is challenging when culturing methods can prove insufficient to isolate each member of the microbiome (Cummings *et al.* 2016). Where other methods of isolating a cyanobacteria-enriched DNA fraction are destructive to the DNA of the heterotrophic microbiome, our new method recovers this DNA (Billi *et al.* 1998). We are currently working to use our method to whole genome sequence other strains in our lab’s culture collection, while further investigations are also needed on BL-A-16 to assess whether the *lim* pathway in this strain is producing prenylated cyanobactins as predicted by the genome sequence analysis.

## Supplementary Material

jkaf135_Supplementary_Data

## Data Availability

Cyanobacterial strains are available upon request. The final polished cyanobacterial genomes are available from GenBank under BioProject PRJNA1048477. The phage sequence was submitted under the accession number PV404223. Additional data, including the Illumina data sets, full metagenomic assemblies, and pycoQC reports, and annotated *lim* pathway in *Limnothrix* sp. BL-A-16 are available on the University of Mississippi Libraries’ eGrove database (https://egrove.olemiss.edu/pharmacy_facpubs/290/), as well as on figshare: https://doi.org/10.25387/g3.29114087. The Supporting Information file contains Flye, DFAST, Geneious Mapping, BLAST, antiSMASH, EPI2ME, TYGS, and OAT analysis results and images of the strains to show cell morphology. Supplemental material available at G3 online.
