# Supplementary material for: Using repeated lysis steps fractionates between heterotrophic and cyanobacterial DNA extracted from xenic cyanobacterial cultures
Source: G3 (Bethesda). 2025 Jun 11;15(8):jkaf135. doi: 10.1093/g3journal/jkaf135 (PMC12341861; doi:10.1093/g3journal/jkaf135)
Supplement: jkaf135_Supplementary_Data [file jkaf135_supplementary_data.pdf]

OXFORD

G3 Genes | Genomes | Genetics

Using Repeated Lysis Steps Fractionates Between Heterotrophic and Cyanobacterial DNA Extracted from Xenic Cyanobacterial Cultures

Alexis D. Wagner[1], Mohammed M. A. Ahmed[1,2], Victoria A. Starks[1] and Paul D. Boudreau[*,1]

[1]Department of BioMolecular Science, School of Pharmacy, University of Mississippi: Faser Hall, University, Mississippi, 38677, United States of America
[2]Department of Pharmacognosy: Al-Azhar University, Cairo, Egypt, 11371

*Corresponding author, e-mail: boudreau@olemiss.edu

Supporting Information - File 1

Keywords: Cyanobacteria; Whole Genome Sequencing; Xenic Cultures; Microbiomes

Legend

- **Genome assembly statistics and analysis**
  - **Table A.** Flye assembly report statistics for BL-A-14.
  - **Table B.** Flye assembly report statistics for BL-A-16.
  - **Table C.** DFAST annotation results of the cyanobacterial chromosome during assembly and polishing.
  - **Table D.** Geneious mapping results for FiltLong processed reads mapped to final assemblies.
  - **Table E.** Megablast search of 1 kb extractions of Illumina draft assembly contigs.
  - **Table F.** AntiSMASH annotation results, draft illumina assemblies versus final assemblies.
  - **Table G.** *lim* pathway comparisons.
  - **Table H.** The EPI2ME classification of filtered reads.
  - **Methods Supplement** Detailed methods for the TYGS analysis.
  - **Figure I.** TYGS analysis of the BL-A-14 genome, the genome-based phylogenetic tree.
  - **Figure J.** TYGS analysis of the BL-A-16 genome, the genome-based phylogenetic tree.
  - **Figure K.** OrthoANI tool analysis of the cyanobacterial chromosomes.
- **Light microscopy images of strains BL-A-14 and BL-A-16**
  - **Figure L** Image of BL-A-14 at 100x in mineral oil.
  - **Figure M** Image of BL-A-16 at 100x in mineral oil.

Table A. Flye assembly report statistics for BL-A-14.

Sequence from Flye Assembly Contig	Length (bp)	Contig Coverage (x Fold)	Circular Contig? (Yes/No)
contig_2	7,179,432	28	Y
contig_3	287,926	28	Y
contig_6	122,116	40	Y
contig_4	66,062	38	Y
contig_44	50,530	30	Y
contig_1	48,739	35	Y
contig_28	24,515	43	Y
contig_31	6,545	6	N
contig_35	6,158	5	N
contig_17	5,278	5	N
contig_29	4,979	6	N
contig_36	4,418	5	N
contig_37	4,314	5	N
contig_39	3,886	3	N
contig_23	3,639	5	N
contig_12	3,549	5	N
contig_10	3,500	1,974	N
contig_19	3,429	5	N
contig_26	3,190	5	N
contig_8	3,006	4	N
contig_24	2,893	6	N
contig_16	2,815	7	N
contig_11	2,770	4	N
contig_41	2,769	5	N
contig_43	2,755	5	N
contig_32	2,664	4	N
contig_9	2,651	5	N
contig_15	2,598	8	N
contig_21	2,580	4	N
contig_33	2,562	4	N
contig_14	2,542	6	N
contig_30	2,486	7	N
contig_40	2,457	3	N
contig_22	2,427	5	N
contig_38	2,421	8	N
contig_13	2,373	4	N
contig_25	2,338	5	N
contig_42	2,333	4	N
contig_34	2,316	4	N
contig_5	2,178	34	Y
contig_27	1,774	5	N
contig_7	1,738	3	N
contig_18	1,214	5	N

Table B. Flye assembly report statistics for BL-A-16.

Sequence from Flye Assembly Contig	Length (bp)	Contig Coverage (x Fold)	Circular Contig? (Yes/No)
contig_1	4,529,414	51	Y
contig_5	1,612,297	35	N
contig_4	1,478,244	35	N
contig_9	1,319,853	35	N
contig_3	741,491	35	N
contig_6	182,609	33	N
contig_11	55,057	101	Y
contig_10	15,887	61	Y
contig_2	5,807	61	N
contig_7	3,529	1,742	N

Table C. DFAST annotation results of the cyanobacterial chromosome during assembly and polishing.

Statistic	*Leptolyngbya* sp. BL-A-14 Chromosome				*Limnothrix* sp. BL-A-16 Chromosome			
	Illumina Draft	Initial from Flye	Medaka Refined	Final from Polypolish	Illumina Draft	Initial from Flye	Medaka Refined	Final from Polypolish
Total Length (bp)	7,013,707	7,179,432	7,183,421	7,186,627	4,017,748	4,529,412	4,532,715	4,539,749
Number of Sequences	526	1	1	1	172	1	1	1
GC Content (%)	50.5	50.5	50.5	50.5	55.2	55.1	55.1	55.2
No. of CDSs	5,853	8,402	7,377	6,233	3,112	6,811	6,277	3,598
No. of rRNA	0	4	4	4	1	4	4	4
No. of tRNA	47	53	53	53	39	45	45	46
No. of CRISPRS	1	1	1	1	4	5	5	5
Coding Ratio (%)	76.4	78.6	80.3	81.9	76.8	73.9	76.2	80.4

This analysis was carried out by the DFAST pipeline using the web-based platform (Tanizawa *et al.* 2018; DNA Data Bank of Japan 2018)

Table D. Geneious mapping results for FiltLong processed reads mapped to final assemblies.

Genome Target for Mapping	*Leptolyngbya* sp. BL-A-14		*Limnothrix* sp. BL-A-16		
	Fraction B (80,600 total reads)	Fraction C (72,412 total reads)	Fraction A (138,058 total reads)	Fraction B (71,060 total reads)	Fraction C (39,807 total reads)
Leptolyngbya sp. BL-A-14	70,729 (88%)	62,321 (86%)	*Not analyzed*	*Not analyzed*	334 (0.84%)*
Limnothrix sp. BL-A-16	*Not analyzed*	*Not analyzed*	48,421 (35%)	53,229 (75%)	33,047 (83%)
Phage of *Limnothrix* sp.	*Not analyzed*	*Not analyzed*	1,680 (1.2%)	777 (1.1%)	417 (1.0%)

*Fraction C reads from *Limnothrix* sp. BL-A-16 were mapped against the genome of *Leptolyngbya* sp. BL-A-14 to assess the rate of false positives in this analysis. With <1% of reads hitting to the other strain's genome, we assessed the false positive rate as low.

Table E. Megablast search of 1 kb extractions of Illumina draft assembly contigs.

Leptolyngbya sp. BL-A-14		*Limnothrix* sp. BL-A-16	
Genus of Top Hit	No. contigs	Genus of Top Hit	No. contigs
Leptolyngbya	617 (92%)	*Limnothrix*	180 (68%)
Streptomyces	8	*Ectopseudomonas*	2
Nocardioides/Nocardioidaceae	8	*Pseudomonas*	79 (30%)
Other (non-cyanobacteria)	35	Other (non-cyanobacteria)	2
No BLAST Hit	1	No BLAST Hit	0
Total	669	Total	263

Table F. AntiSMASH annotation results, draft illumina assemblies versus final assemblies.

AntiSMASH Results	*Leptolyngbya* sp. BL-A-14 Illumina Draft	Final	*Limnothrix* sp. BL-A-16 Illumina Draft	Final
Total Hits	11	10	4	4
terpene/resorcinol	5	5	2	2
NRPS/NRPS-like	5	2	0	0
phenazine	1	1	0	0
transAT-pks	0	0	1	1
cyanobactin	0	0	1	1
proteusin	0	1	0	0
NRPS,NRPS-like,T1PKS	0	1	0	0
Hits on Contig Edges	11	0	2	0

Table G. *lim* pathway comparisons.

Gene	Pairwise %ID (gene/predicted protein)	Annotation	
		BL-A-16	CACIAM 69d
limH	97.7 / 99.5	Type II secretion system F gamily protein	Type II secretion system F gamily protein
limI	98.8 / 100	TypeIV pilus twitching motility protein PilT	TypeIV pilus twitching motility protein PilT
limJ	96.9 / 99.6	TypeII/IV secretion system protein	GspE/PulE family protein
limK	96.3 / 96.3	GrpE nucleotide exchange factor	GrpE nucleotide exchange factor
limL	95.6 / 97.9	DnaK molecular chaperone	DnaK molecular chaperone

Table H. The EPI2ME classification of filtered reads.

Superkingdom	Kingdom	Phylum	BLA14-B	BLA14-C	BLA16-A	BLA16-B	BLA16-C
Unclassified	Unknown	Unknown	75,112	63,558	62,596	56,458	33,836
Bacteria	Bacteria	Cyanobacteriota	1,973	2,954	2,232	1,938	1,322
Bacteria	Bacteria	Acidobacteriota	3	10	16	7	4
Bacteria	Bacteria	Actinomycetota	1,144	1,397	402	183	120
Bacteria	Bacteria	Aquificota	2	3	0	1	0
Bacteria	Bacteria	Armatimonadetes	0	0	2	0	2
Bacteria	Bacteria	Bacillota	165	273	159	80	89
Bacteria	Bacteria	Bacteroidota	58	89	66	67	41
Bacteria	Bacteria	Chlamydiota	1	0	1	0	1
Bacteria	Bacteria	Chlorobiota	0	3	7	6	10
Bacteria	Bacteria	Chloroflexota	10	12	4	1	0
Bacteria	Bacteria	Chrysiogenetes	0	1	0	0	0
Bacteria	Bacteria	Coprothermobacterota	0	0	1	0	0
Bacteria	Bacteria	Deferribacteres	1	0	0	0	0
Bacteria	Bacteria	Deinococcota	3	3	4	4	3
Bacteria	Bacteria	Fusobacteriota	1	1	0	1	0
Bacteria	Bacteria	Gemmatimonadota	0	1	1	0	1
Bacteria	Bacteria	Ignavibacteriae	0	1	0	0	0
Bacteria	Bacteria	Kiritimatiellaeota	0	0	0	1	0
Bacteria	Bacteria	Mycoplasmatota	1	1	2	1	1
Bacteria	Bacteria	Nitrospirota	2	1	2	1	1
Bacteria	Bacteria	Planctomycetota	12	26	15	7	10
Bacteria	Bacteria	Pseudomonadota	1,507	3,287	69,691	11,554	3,922
Bacteria	Bacteria	Spirochaetota	2	4	3	4	0
Bacteria	Bacteria	Synergistota	0	0	0	1	0
Bacteria	Bacteria	Thermomicrobiota	0	0	0	1	0
Bacteria	Bacteria	Thermotogota	1	4	0	0	1
Bacteria	Bacteria	Verrucomicrobiota	4	3	17	19	8
Archaea	Archaea	Ca. Thermoplasmatota	0	2	1	0	0
Archaea	Archaea	Crenarchaeota	1	2	1	0	0
Archaea	Archaea	Euryarchaeota	17	23	27	15	11
Archaea	Archaea	Thaumarchaeota	0	1	0	0	0
Viruses	Orthornavirae	Kitrinoviricota	0	1	0	1	0
Viruses	Orthornavirae	Negarnaviricota	0	0	0	1	0
Viruses	Orthornavirae	Pisuviricota	0	1	0	0	0
Viruses	Heunggongvirae	Uroviricota	4	4	3	1	0
Viruses	Viruses Incertae sedis	Viruses Incertae sedis	0	0	0	1	0
Eukaryota	Metazoa	Chordata	66	94	69	27	28

Methods Supplement: Detailed methods for the TYGS analysis.

The TYGS analysis was carried out on their web-based platform (https://tygs.dsmz.de/), detailed methods for the creation of the generation of the phylogenetic trees are provided below, unmodified, from that resource:

The genome sequence data were uploaded to the Type (Strain) Genome Server (TYGS), a free bioinformatics platform available under https://tygs.dsmz.de, for a whole genome-based taxonomic analysis (Meier-Kolthoff and Göker 2019). The analysis also made use of recently introduced methodological updates and features (Meier-Kolthoff *et al.* 2022). Information on nomenclature, synonymy and associated taxonomic literature was provided by TYGS's sister database, the List of Prokaryotic names with Standing in Nomenclature (LPSN, available at https://lpsn.dsmz.de) (Meier-Kolthoff *et al.* 2022). The results were provided by the TYGS on 2025-03-19. The TYGS analysis was subdivided into the following steps:

Determination of closely related type strains. Determination of closest type strain genomes was done in two complementary ways: First, all user genomes were compared against all type strain genomes available in the TYGS database via the MASH algorithm, a fast approximation of intergenomic relatedness (Ondov *et al.* 2016), and, the ten type strains with the smallest MASH distances chosen per user genome. Second, an additional set of ten closely related type strains was determined via the 16S rDNA gene sequences. These were extracted from the user genomes using RNAmmer (Lagesen *et al.* 2007) and each sequence was subsequently BLASTed (Camacho *et al.* 2009) against the 16S rDNA gene sequence of each of the currently 22776 type strains available in the TYGS database. This was used as a proxy to find the best 50 matching type strains (according to the bitscore) for each user genome and to subsequently calculate precise distances using the Genome BLAST Distance Phylogeny approach (GBDP) under the algorithm 'coverage' and distance formula d5 (Meier-Kolthoff *et al.* 2013). These distances were finally used to determine the 10 closest type strain genomes for each of the user genomes.

Pairwise comparison of genome sequences. For the phylogenomic inference, all pairwise comparisons among the set of genomes were conducted using GBDP and accurate intergenomic distances inferred under the algorithm 'trimming' and distance formula d5 (Meier-Kolthoff *et al.* 2013). 100 distance replicates were calculated each. Digital DDH values and confidence intervals were calculated using the recommended settings of the GGDC 4.0 (Meier-Kolthoff *et al.* 2022, 2013).

Phylogenetic inference. The resulting intergenomic distances were used to infer a balanced minimum evolution tree with branch support via FASTME 2.1.6.1 including SPR postprocessing (Lefort *et al.* 2015). Branch support was inferred from 100 pseudo-bootstrap replicates each. The trees were rooted at the midpoint (Farris 1972) and visualized with PhyD3 (Kreft *et al.* 2017).

Type-based species and subspecies clustering. The type-based species clustering using a 70% dDDH radius around each of the 19 type strains was done as previously described (Meier-Kolthoff and Göker 2019). The resulting groups are shown in Table 1 and 4. Subspecies clustering was done using a 79% dDDH threshold as previously introduced (Meier-Kolthoff *et al.* 2014).

Type-based species and subspecies clustering. The resulting species and subspecies clusters are listed in Table 4, whereas the taxonomic identification of the query strains is found in Table 1. Briefly, the clustering yielded 20 species clusters and the provided query strains were assigned to 1 of these. Moreover, user strains were located in 1 of 20 subspecies clusters.

Figure I. TYGS analysis of the BL-A-14 genome, the genome-based phylogenetic tree.

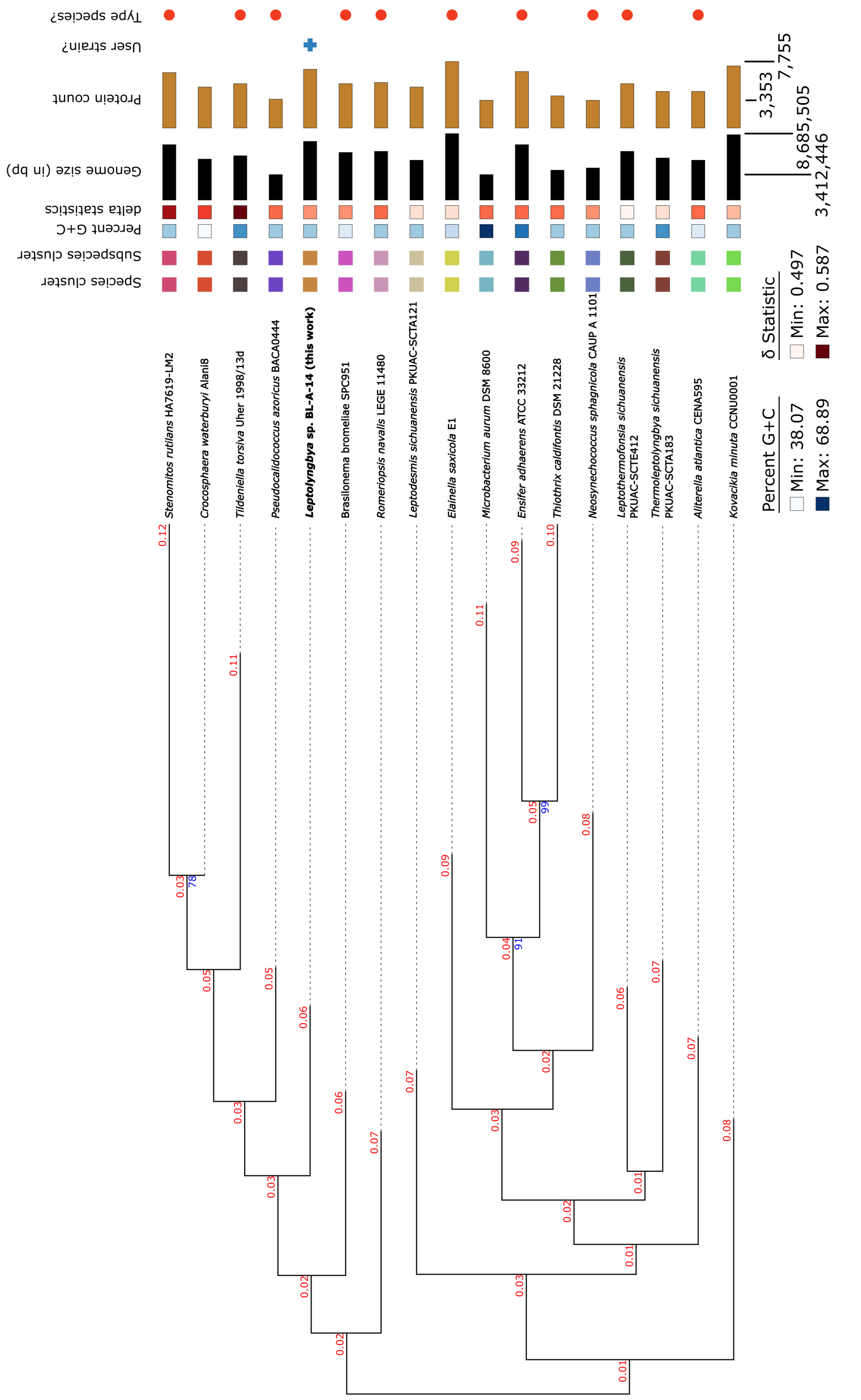

Genome-based tree made using the TYGS platform, with additional labels appended for clarity. Tree inferred with FastME 2.1.6.1 (Lefort *et al.* 2015) from GBDP (Genome BLAST Distance Phylogeny) distances calculated from genome sequences. The branch lengths are scaled in terms of GBDP distance formula d5 (represented in red numbers). The numbers above branches are GBDP pseudo-bootstrap support values > 60% from 100 replications (represented in blue numbers), with an average branch support of 32.0%. The tree was rooted at the midpoint (Farris 1972).

Figure J. TYGS analysis of the BL-A-16 genome, the genome-based phylogenetic tree.

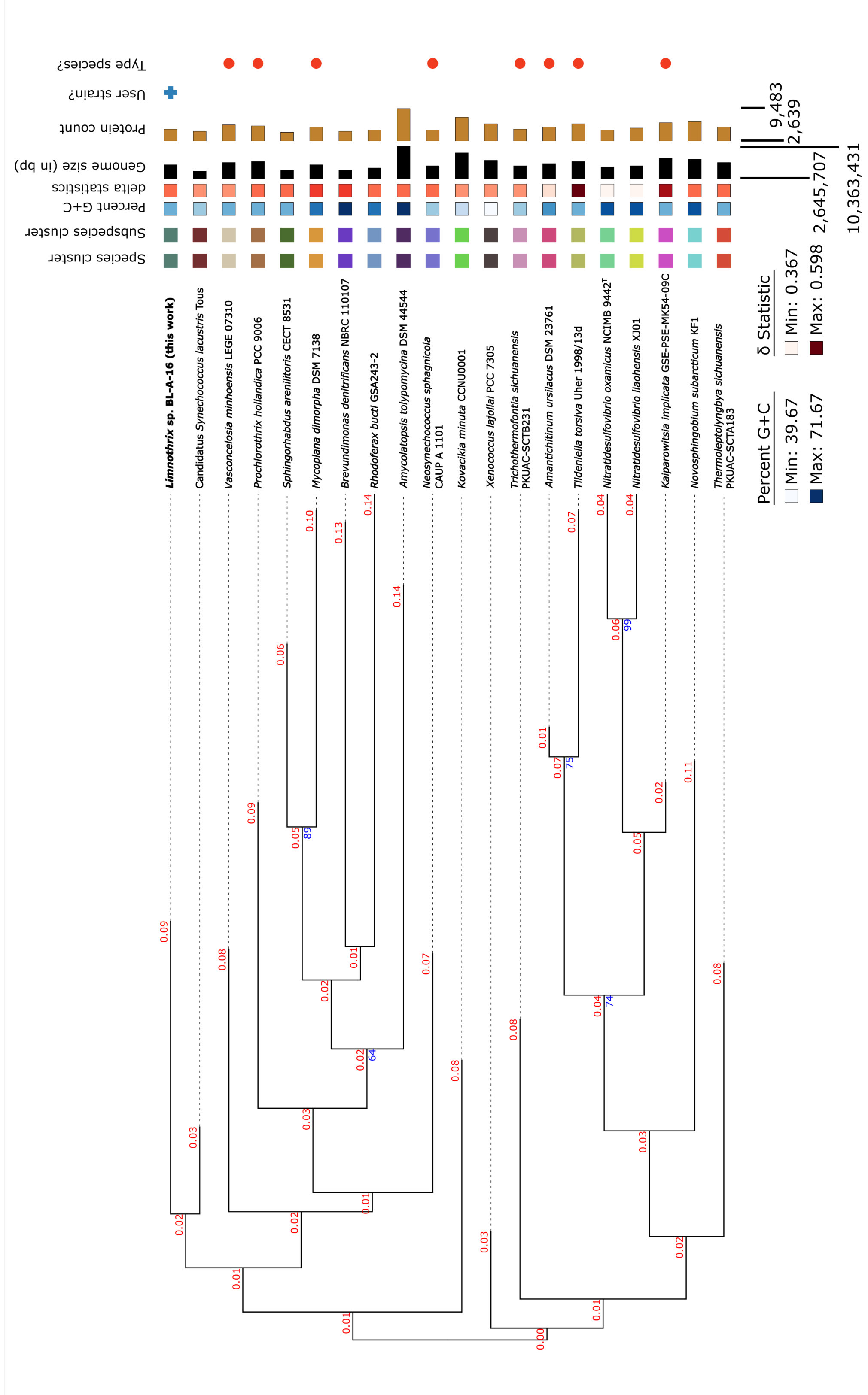

Genome-based tree made using the TYGS platform, with additional labels appended for clarity. Tree inferred with FastME 2.1.6.1 (Lefort *et al.* 2015) from GBDP (Genome BLAST Distance Phylogeny) distances calculated from genome sequences. The branch lengths are scaled in terms of GBDP distance formula d5 (represented in red numbers). The numbers above branches are GBDP pseudo-bootstrap support values >60% from 100 replications (represented in blue numbers), with an average branch support of 39.2%. The tree was rooted at the midpoint (Farris 1972).

Figure K. OrthoANI tool analysis of the cyanobacterial chromosomes.

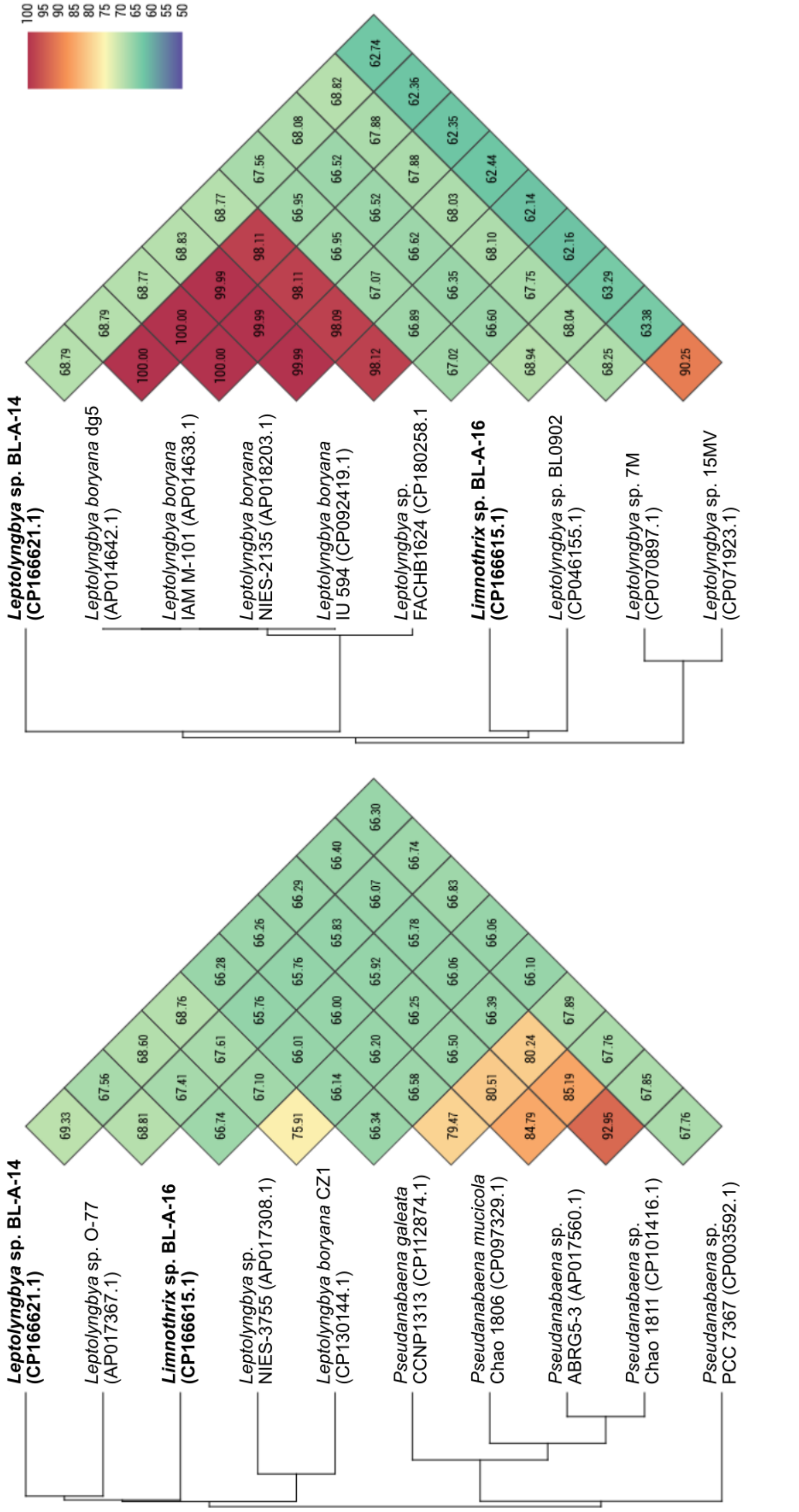

Analysis performed with the OrthoANI Tool version 0.93.1 (Lee *et al.* 2016).

Figure L. Image of BL-A-14 at 100x in mineral oil.

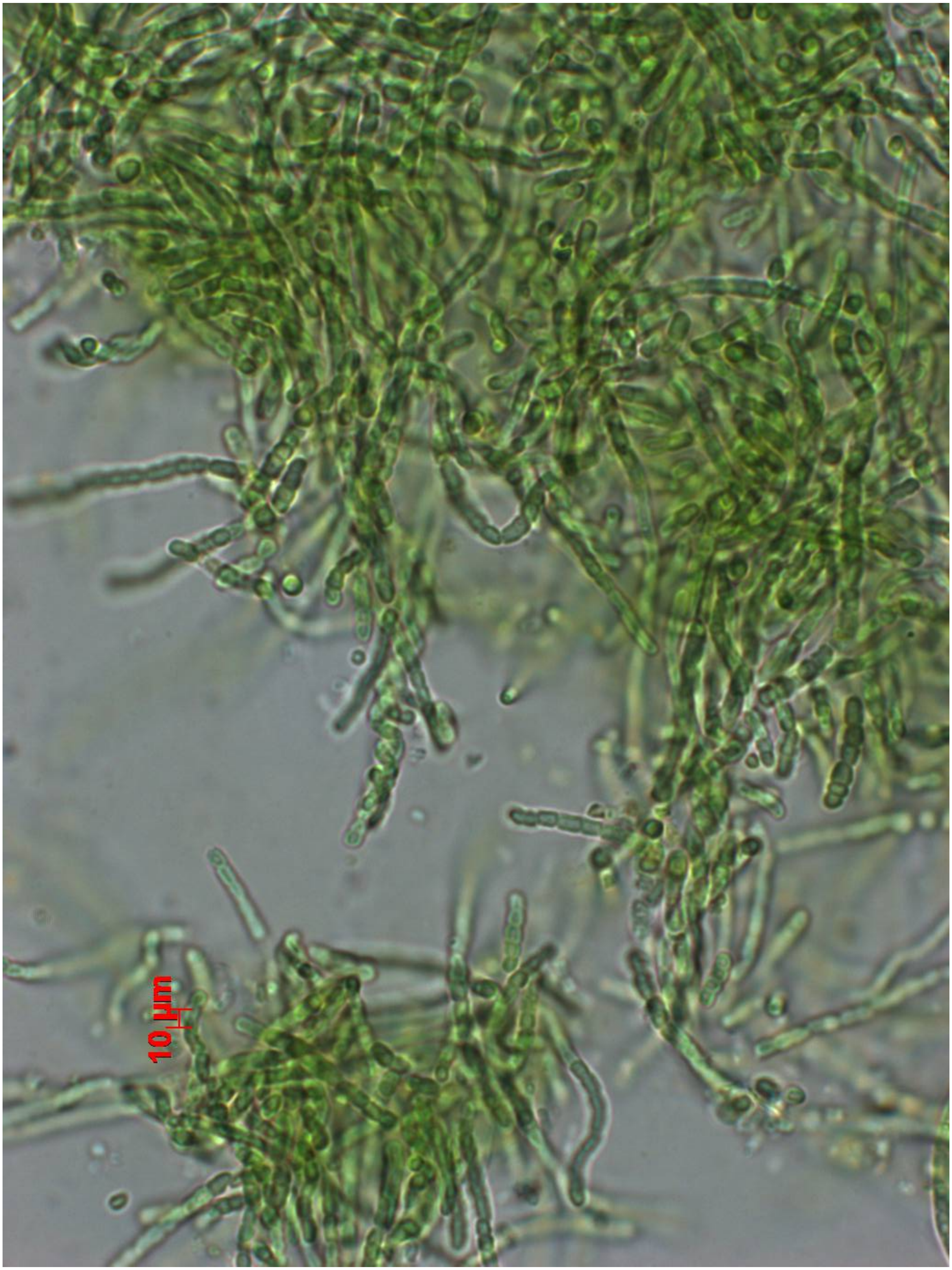

Figure M. Image of BL-A-16 at 100x in mineral oil.

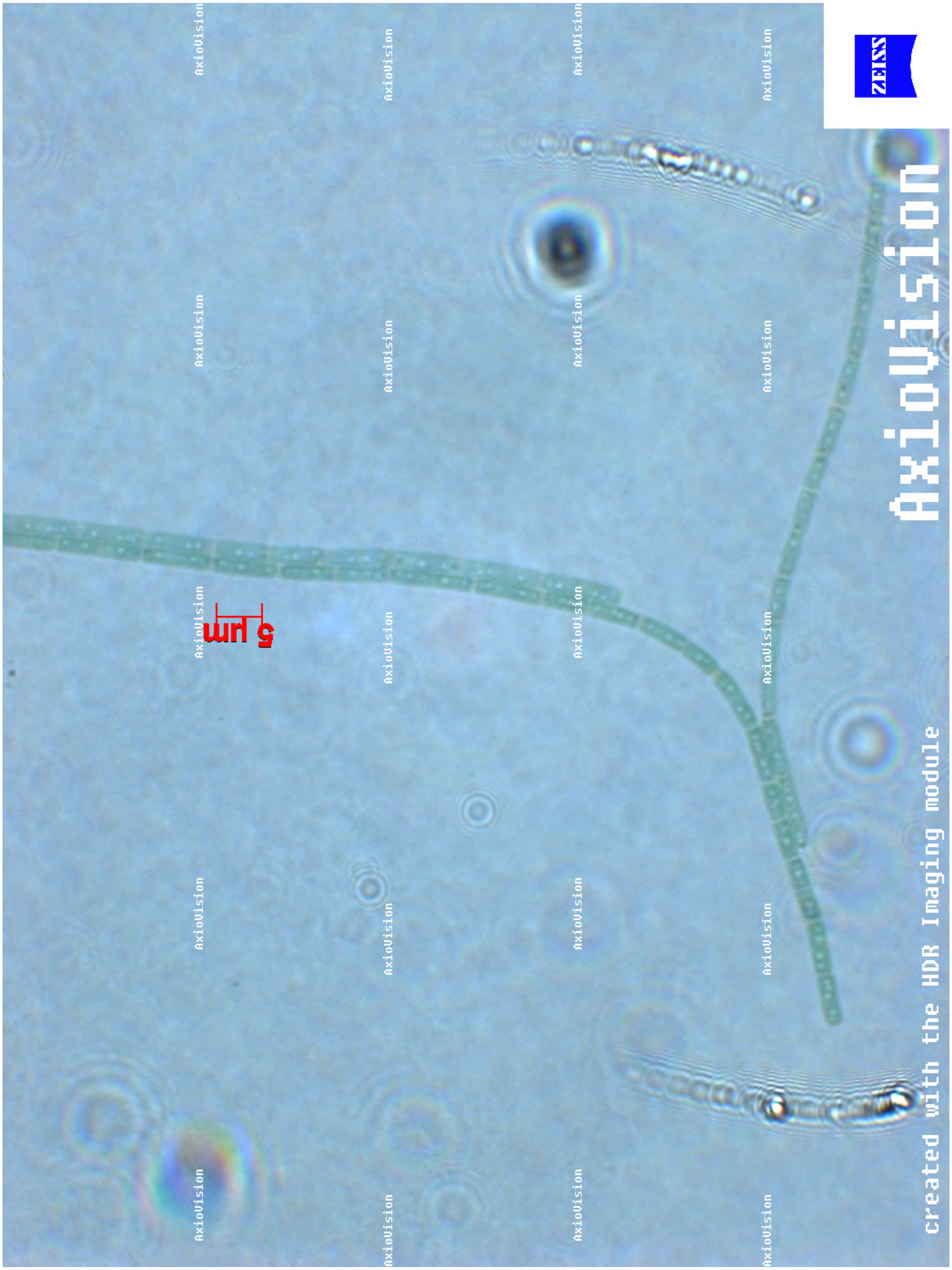

Literature cited

Camacho C, Coulouris G, Avagyan V, Ma N, Papadopoulos J, Bealer K, Madden TL. 2009. BLAST+: architecture and applications. BMC Bioinformatics. 10:421.

DNA Data Bank of Japan. 2018. DFAST. https://dfast.ddbj.nig.ac.jp/. Accessed: 2023-09-25.

Farris JS. 1972. Estimating phylogenetic trees from distance matrices. The American Naturalist. 106:645–668.

Kreft Å, Botzki A, Coppens F, Vandepoele K, Van Bel M. 2017. Phyd3: a phylogenetic tree viewer with extended phyloxml support for functional genomics data visualization. Bioinformatics. 33:2946–2947.

Lagesen K, Hallin P, Rødland EA, Stærfeldt HH, Rognes T, Ussery DW. 2007. Rnammer: consistent and rapid annotation of ribosomal rna genes. Nucleic Acids Research. 35:3100–3108.

Lee I, Kim YO, Park SC, Chun J. 2016. OrthoANI: An improved algorithm and software for calculating average nucleotide identity. International Journal of Systematic and Evolutionary Microbiology. 66:1100–1103.

Lefort V, Desper R, Gascuel O. 2015. Fastme 2.0: A comprehensive, accurate, and fast distance-based phylogeny inference program. Molecular Biology and Evolution. 32:2798–2800.

Meier-Kolthoff JP, Auch AF, Klenk HP, Göker M. 2013. Genome sequence-based species delimitation with confidence intervals and improved distance functions. BMC Bioinformatics. 14:60.

Meier-Kolthoff JP, Carbasse JS, Peinado-Olarte RL, Göker M. 2022. TYGS and LPSN: a database tandem for fast and reliable genome-based classification and nomenclature of prokaryotes. Nucleic acids research. 50:D801–D807.

Meier-Kolthoff JP, Göker M. 2019. TYGS is an automated high-throughput platform for state-of-the-art genome-based taxonomy. Nature Communications. 10.

Meier-Kolthoff JP, Hahnke RL, Petersen J, Scheuner C, Michael V, Fiebig A, Rohde C, Rohde M, Fartmann B, Goodwin LA *et al*. 2014. Complete genome sequence of DSM 30083T, the type strain (U5/41T) of *Escherichia coli*, and a proposal for delineating subspecies in microbial taxonomy. Standards in Genomic Sciences. 9:2.

Ondov BD, Treangen TJ, Melsted P, Mallonee AB, Bergman NH, Koren S, Phillippy AM. 2016. Mash: fast genome and metagenome distance estimation using MinHash. Genome Biology. 17:132.

Tanizawa Y, Fujisawa T, Nakamura Y. 2018. DFAST: A flexible prokaryotic genome annotation pipeline for faster genome publication. Bioinformatics. 34:1037–1039.
